# Multifactorial background of acute sports injuries in young Japanese athletes—a semi-structured interview study

**DOI:** 10.3389/fspor.2026.1813583

**Published:** 2026-05-26

**Authors:** Yukiko Kimotsuki, Issei Ogasawara, Susumu Iwasaki, Kanto Nagai, Kyohei Nishida, Ryosuke Kuroda, Ken Nakata

**Affiliations:** 1 Department of Health and Sport Sciences, Graduate School of Medicine, The University of Osaka, Osaka, Japan; 2 Department of Health and Human Performance, Fort Lewis College, Durango, CO, United States; 3 Department of Orthopaedic Surgery, Kobe University Graduate School of Medicine, Hyogo, Japan

**Keywords:** athletic injuries, cognitive, psychology, risk factors, social

## Abstract

**Background:**

Although sports injuries occur because of inappropriate physical movement, the influence of multiple factors, such as psychological and social factors, on their occurrence remains poorly understood.

**Methods:**

This semi-structured interview study included 10 athletes. The semi-structured interviews explored participants’ perceptions of the physical, psychological, cognitive, social, and situational factors associated with their injury. Their conversations were audio recorded, transcribed, and qualitatively analyzed by three experts using the Steps for Coding and Theorization method (SCAT).

**Results:**

The SCAT extracted 14 key themes, which can be categorized into seven meta-factors as potential factors underlying sports injuries. Notably, athletes often misjudge their physical state and overlook early warning signs of discomfort, while lacking sufficient behavioral knowledge on how to control and prevent risk. Additionally, elevated stress and excessive motivation triggered risk-taking behaviors and persistence, despite fatigue. A lack of external guidance and feedback appeared to allow maladaptive routines to continue. Situational factors, such as unfamiliar tactics combined with limited technical adaptability, reduced their ability to adjust movement behavior and ultimately increased vulnerability to injury.

**Conclusion:**

Exploration of meta-factors and key themes revealed that sports injuries are influenced by a multitude of factors, irrespective of athletes’ awareness.

## 1 Introduction

Prevention of sports injuries remains a complex issue with substantial societal and clinical implications (1). Severe injuries threaten athletes’ well-being and careers and may adversely impact team performance (2, 3). Moreover, such injuries increase the risk of re-injury and long-term disability. Sports injuries occur within complex and dynamic contextual environments (4). Understanding the multifaceted factors that contribute to injuries, including biomechanical, physiological, psychological, social, and environmental factors, is essential for effective prevention (4–6). Although recent research has broadened the scope of this investigation (4), the precise relationship between injury occurrence and these diverse risk factors remains inadequately understood. Therefore, there is an urgent need for a more comprehensive and context-sensitive understanding of the mechanisms underlying sports injuries.

One of the prominent frameworks addressing psychological contributors to sports injuries is the Stress-Injury Model (7). According to this model, stressors such as life events, everyday hassles, competitive demands, and anxiety may trigger physiological and cognitive responses, namely increased muscular tension, reduced attentional breadth, and greater susceptibility to distraction, which collectively increase the likelihood of injury.

Cognitive factors also play a critical role in injury risk (8). For instance, impaired cognitive performance, particularly during tasks requiring rapid decision making, has been associated with a greater likelihood of injury. Although a substantial portion of the existing evidence is derived from survey-based studies, experimental findings indicate that impairments in working memory and reaction time may increase musculoskeletal injury risk (9, 10). However, how these cognitive limitations manifest in real-world sports settings remains poorly understood.

Social factors such as the quality of relationships with teammates and coaches may contribute to injury risk. Research has shown that interpersonal stress within a team environment is associated with a higher likelihood of sustaining injuries (11). The concept of motivational climate in Achievement Goal Theory refers to the social environment created by coaches and teammates, which influences how individuals pursue their goals (12). Although a previous quantitative study using a questionnaire did not find a direct relationship between motivational climate and injury risk (11), the observed connection between social factors and injury occurrence suggests that a qualitative approach could reveal motivational climate as a possible factor contributing to injury onset. Furthermore, non-sports-related stressors (arising from major life events) contribute to increased vulnerability to injury (13). Although some studies have investigated social support during injury recovery (14), the influence of the social context on injury causation has not been sufficiently explored.

Situational factors (the immediate circumstances surrounding injury events) vary widely, even within the same sport. Factors such as timing within the competitive season or specific match conditions affect the likelihood of injury, such as hamstring injuries among soccer players (13).

Taken together, sports injuries can be understood as a result of a complex interplay between multiple diverse factors. Systematically integrating and elucidating these factors based on athletes’ lived experiences is essential for achieving a comprehensive understanding of how sports injuries occur. Accordingly, the present study makes a highly meaningful contribution by aiming to clarify the overall structure of the multifaceted background factors underlying sports injuries.

To achieve this aim, a qualitative research approach was adopted, as it enables the generation of contextual and novel insights into athletes’ experiences that cannot be fully captured through quantitative methods. The purpose of this study was to elucidate the overall structure of the background factors contributing to sports injuries based on the narratives of injured athletes. The findings of this study are expected to enhance understanding of injury mechanisms and provide a foundation for developing new prevention strategies.

## 2 Methods and materials

### 2.1 Participant recruitment

Participants who previously participated in a web-based survey examining the psychological factors associated with sports injuries were recruited for this study (15). Among these respondents, individuals who expressed a willingness to participate in a follow-up interview and had experienced a sports-related injury requiring at least a 1-week suspension from athletic activities were considered eligible. This threshold aligns with the International Olympic Committee (IOC) consensus statement, in which injuries resulting in seven or more days of time loss are classified as moderate or greater severity (16). Based on this internationally recognized definition, the present study targeted athletes who had experienced injuries of at least moderate severity, consistent with the study's aim of generating foundational knowledge on the multifactorial mechanisms underlying moderate-to-severe sports injuries.

Twenty-six eligible athletes were contacted via email, which included an explanation of the study's purpose and procedure. Of them, 17 agreed to participate and were subsequently interviewed. Written informed consent was obtained from all 17 participants before the interviews.

Data from 10 athletes diagnosed with acute sports-related injuries were included; however, seven participants who could not recall a specific diagnosis or who reported chronic sports injuries were excluded. Notably, the final analytic sample consisted predominantly of athletes with severe injuries, including anterior cruciate ligament (ACL) tears and other conditions requiring prolonged interruption of sport participation. The study protocol and interview procedures were reviewed and approved by the Ethics Committee of the University of Osaka Hospital (Approval No. 21216). Information on the participants is presented in Table 1.

**Table 1:** Participant information.

Participant	Sex	Age at time of injury	Sport	Level of competition	Diagnosis
#1	Female	17/21	Lacrosse	Local	ACL tear (Both legs)
#2	Male	15 /17	Handball	National	Left lateral meniscus injury(twice)
#3	Female	18	Lacrosse	Local	Left ACL tear, Left meniscus injury
#4	Male	20/20	Handball	Local	Right ACL tear, Right Meniscus injury(twice)
#5	Female	16 /17	Basketball	Prefectural	Right ACL tear(twice)
#6	Female	21	Ultimate	Local	Left ACL injury
#7	Female	21	Handball	Local	Left ACL tear
#8	Male	19	Gymnastics	Intermediate	Shoulder dislocation
#9	Female	21	Lacrosse	Local	Left ACL tear
#10	Female	20/20	Lacrosse	Local	Left ACL tear

ACL, Anterior cruciate ligament.

### 2.2 Data collection

Semi-structured interviews were conducted by the lead author (Y.K) between April 2021 and October 2023, either in person or remotely, via Zoom. Each interview lasted approximately 30–40 minutes and was guided by a semi-structured interview protocol developed by the research team.

The initial development of the interview guide was informed by the framework proposed by Kallio et al. (17), and the conceptual basis was derived from the Risk Structure Model of sports injury proposed by Ogasawara et al. (18, 19) This concept posits that sports injuries result from a sequence of interconnected risks initiated by athletes’ psychological and physical factors. These factors interact with affordances from the environment, perceptual and cognitive elements, and poor biomechanics, ultimately leading to mechanical processes that cause tissue failure. The draft of the interview guide was reviewed by three experts [a biomechanics researcher and certified athletic trainer [I.O.], a sports psychology researcher [S.I.], and a sports medicine researcher [Y.K.]]. Dr. Issei Ogasawara [I.O.], who has over twenty years of research and practical experience in sports biomechanics and athletic training, contributed to the review. Dr. Susumu Iwasaki [S.I.], with more than twenty years of experience in sports psychology research and athlete mental support, also participated in the review. Mrs. Yukiko Kimotsuki [Y.K.], with ten years of clinical experience as a registered nurse and six years of research experience in sports medicine, contributed to the development and refinement of the interview guide. The interview guide comprised 10 components: 1) basic competition information; 2) an injury history; 3) the situation at the time of the injury; 4) reasons for selecting the play that led to the injury; 5) positional relationships with the opponent and any equipment at the time of injury; 6) body movements executed at the time of the injury; 7) participant's perceptions of the causes of the injury; 8) considerations for future injury prevention strategies; 9) an advice to others on injury prevention; and 10) values and priorities guiding the participant's approach to sports.

Pilot interviews were conducted with three athletes—one regional-level, one national-level, and one professional—to assess the clarity, sequencing, and appropriateness of the questions, as well as the feasibility of the interview duration. The same procedures and ten components were used during the pilot interviews. Following pilot testing, the interview protocol was refined. While the original 10 components were retained, additional probing questions were added to items 1), 5), and 6). These modifications were made because some pilot participants found the original questions difficult to answer or provided insufficient detail, particularly about the contextual aspects of the injury event.

To promote thoughtful responses, a finalized interview guide was sent to the participants via email 1 week before the interview. The participants were instructed to read the guide in advance and confirm their understanding before the interview commenced. To safeguard participants’ well-being, they were informed that the interview would be paused immediately if signs of psychological distress or physical discomfort were observed while recalling their injury. The interviews’ audio recordings were obtained using the Zoom platform's integrated recording function to ensure accurate data capture for subsequent analyses.

### 2.3 Interview data analysis

Steps for Coding and Theorization (SCAT) was employed as a qualitative analytic technique designed for the systematic analysis of narrative data and generation of theoretically meaningful insights through a stepwise coding and theorization process (20). SCAT was selected for several reasons. First, the present study aimed to explore multifactorial and context-dependent mechanisms underlying sports injuries based on participants’ narratives. The structured yet flexible analytic framework of SCAT, which progresses from key word extraction to conceptualization and theorization, is well suited to capturing such complex and interrelated factors. Second, SCAT is particularly effective for studies with relatively small sample sizes, as it enables rigorous and transparent theory construction without requiring large datasets. This characteristic aligns with the exploratory nature of the present study. In addition, because the interview data were collected in Japanese, SCAT—originally developed in Japan—was considered appropriate for preserving the semantic nuances of participants’ narratives during analysis.

Briefly, the SCAT comprised four coding steps and a theorization process. In the first step, keywords relevant to the research context were extracted from the narrative data. In the second step, these keywords were substituted with alternative terms that were not explicitly present in the original data. In the third step, external concepts were introduced to explain the extracted terms, thereby expanding the interpretive framework. In the fourth step, all terms—including those derived from the original data and those added during the expansion process—were grouped according to semantic similarity, and overarching conceptual labels (“themes”) were generated. Finally, the theorization process, in which the themes were integrated into a coherent theoretical explanation grounded in the participants’ narratives. A general overview of the coding procedures is described in a previous study (20).

In this study, the SCAT procedure was conducted by three experts, who carefully progressed through each step while reaching a consensus among the three members at all stages. Qualitative data analysis software was not used during the analytic process; all coding and theorization procedures were conducted manually by the research team. A representative example of the coding process was summarized in Supplementary Table S1. First, the transcribed interview data were broken down into individual words, from which semantically significant terms related to sports injuries, reflecting the content of the interview questions, were extracted. In this study, “semantically significant” referred to words that, depending on the question posed to the participant, met one or more of the following criteria:

- Nouns directly represent the sports injury itself.
- Verbs, gerunds, adverbs, or adjectives describing bodily movements or states during injury onset.
- Nouns and adjectives expressing the participant's perceptual or psychological state.
- Nouns and adjectives characterize the social environment surrounding the participant.

For example, from the original narrative that stated “I was feeling relatively well in March” and “I’m not sure… I feel like I couldn't have prevented the first injury, but for the second one, I probably shouldn't have pushed myself. My knee felt a bit unstable”, the keywords of “feeling well” and “knee instability” were extracted.

In the second step, the extracted keywords in the previous step were replaced with alternative terms that had similar meanings but did not explicitly appear in the original narratives. For instance, “feeling well” was replaced with “good condition.” While the term “good condition” did not appear in the narrative data, its introduction expanded the semantic space of the term group and contributed to the derivation of a more generalizable theory. This replacement process was conducted with careful deliberation by the research team to ensure that the new terms remained within the original data semantic range.

In the third step, explanatory concepts external to the original data were introduced to interpret the substituted terms. For example, “good condition” was expanded to “subjective sense of well-being” and “knee instability” was expanded to “awareness of knee discomfort.” These additions broadened the semantic space while remaining within the interpretive range of the original narratives.

In the fourth step, the full set of concepts, comprising those derived from the third step, was grouped based on semantic similarity. Each group was assigned a conceptual label, referred to as a “theme.” Thematic categories were generated by grouping the concepts identified up to Step 4 based on their semantic similarity. This grouping process, as well as the labeling of each group with appropriate thematic titles, was conducted carefully through frequent discussions among three experts. To further ensure validity, parallel reviews were conducted by additional researchers who were not involved in the initial analytical process.

Within this constructivist semantic analysis, concepts that appeared to have limited direct relevance to sports injuries were not dismissed if they were commonly shared across multiple athletes’ narratives or emerged repeatedly within the context of a single interview. Such contextually salient concepts were retained and selected as themes. Their relevance to sports injuries was subsequently examined and interpreted in relation to existing literature. For example, the concepts of “subjective sense of well-being” and “awareness of knee discomfort” derived from a specific context were integrated into the theme “the mismatch between perceived and actual physical condition.” Additionally, to explore higher-order structure of the identified themes, they were grouped based on meaning to derive an overarching “Meta-factor.”

Finally, in the theorization process, a succinct theoretical description and storyline of sports injuries were articulated by integrating the expanded themes’ semantic content with contextual information from the participants’ narratives. In SCAT, the storyline refers to a concise theoretical explanation that integrates the expanded themes and contextual elements of participants’ narratives to illustrate the underlying mechanism through which the phenomenon occurs.

### 2.4 Sample size and semantic saturation

The sample size of the present study (10 participants) was determined based on semantic saturation of the data. During the analytical process, after the narratives of approximately five to six participants had been analyzed, no new conceptual meanings, semantic dimensions, or interpretive extensions were identified, and repetition of semantic content became evident. This was interpreted as indicating that semantic saturation had been achieved (21). This judgment was made based on consensus among three experts.

### 2.5 Reliability and confirmability

Multiple strategies were used to enhance the reliability and confirmability of the analysis. First, the three experts independently reviewed the verbatim transcripts and conducted all four SCAT steps before discussion. This independent process reduced the risk of premature consensus and helped minimize the influence of individual preconceptions. Subsequently, consensus meetings were held to compare and integrate the codes, concepts, and themes generated at each analytic step. When discrepancies arose, they were resolved through discussion with reference to the study's theoretical framework, the narrative context, and relevant prior literature.

To further enhance rigor, the experts explicitly discussed their disciplinary perspectives and assumptions throughout the process to strengthen reflexivity. In addition, peer debriefing was conducted with qualitative research experts who were not involved in the study. An audit trail was also maintained to document major analytic decisions, including the treatment of narratives that did not fit the final thematic structure.

Verification of the analytic results with the participants (member checking) was not conducted because a substantial period had elapsed between the interviews and the completion of the analysis, making participant follow-up difficult owing to changes in affiliation and raising concerns about additional participant burden. Instead, credibility and confirmability were supported through analyst triangulation, peer debriefing, reflexive discussion, and the audit trail.

## 3 Results

Fourteen themes emerged from the interview analysis (Tables 2, 3). After a thorough assessment by three experts, they were systematically organized into seven overarching meta-factors: physical factor (perceived physical related), educational factor, psychological factor, cognitive factor, social factor, situational factor, and skill and body movement factor.

**Table 2:** Themes and theoretical descriptions were extracted from the interview data using SCAT.

Meta-factor	Theme	Theoretical descriptions
Physical factor (Perceived physical-related)	The mismatch between perceived and actual physical condition	Athletes recognized that they were in a good physical/mental condition, however, they were actually overworked and tired. Athlete was aware that he was in good physical and mental shape, but he ignored the discomfort and instability in his knees. Athlete thought that her physical and mental condition was not bad; however, she was afraid of any movement since her rehabilitation was not successful (Second injury case). Athlete felt decreased physical strength and poor technique due to a long hiatus from the match; she trained very hard for her upcoming retirement match. There was a possibility of misperception of the actual physical condition. Before the match, the athlete's physical condition was poor due to lack of sleep, but once the match started, he felt in good condition. Athlete was in a decreased physical condition after the university examination; however, he felt that his physical condition was the same as usual.
	Ignoring injury warning signs	Athlete was concerned about instrumental factors, but she didn't take any special measures. There were injury warning signs, but no precautions were taken. Since others pointed out the possibility of injury, the athlete felt the risk of injury. However, she did not take any special action. Athlete recognized his side-to-side difference in lower-limb muscle strength might be a risk of injury, but he did not take any special action to solve the problem.
Educational factor	Limited knowledge of injury and prevention	Limited information regarding injury prevention and self-regulation. Limited knowledge about the mechanism of sports injuries. Athletes did not know about self-regulation. The injury was unexpected, although the athlete felt some knee discomfort. Athlete did not know about the injury itself at the first injury. The athlete did not know how to prevent re-injury at the second injury. The team had no coaches who could instruct safe sport play. Luck also plays a role in the occurrence of injuries. Athletes, coaches, and trainers do not know how to control reinjury risks.
Psychological factor	High-stress condition	Before the injury, the athlete was in a high-stress and disharmonious state, but the athlete didn't know about it. Athletes were nervous about returning to the match during the rehabilitation period, and she also felt fear about the movement in the match. The leadership role heightened the athlete's sense of responsibility, making her particularly sensitive to the possibility of mistakes. During the match, there was pressure from a certain teammate who was extremely impatient. Athlete was intensely driven to earn a place in the starting lineup.
	Elevated motivation levels	Athlete chose a more challenging and unfamiliar play, resulting in an injury. Athlete had a high motivation for growth. Athlete had a strong desire to score and was focused on playing an active role. Athlete strongly expected his success. Before the first time injury, the athlete was so excited to be selected as a starting member soon. At the second time injury, she was desperate to become a starting member again.
Cognitive factor	Narrowing of attention	There was insufficient focus, poor awareness of the surroundings, and limited spatial understanding. Since the athlete was aware of her poor skills and tactics, she had low confidence in decision-making. Driven by a strong desire to score, the athlete's attention became overly concentrated, leading to reduced situational awareness and suboptimal play choices.
	Overconfidence	Overconfidence in their play led to injuries when their decisions turned out to be wrong. Athlete thought her decision was correct at the time of injury. Athlete had greater overconfidence in their physical ability and self-assessment than others. Despite recognizing the inappropriateness of the decision that led to the injury, the athlete tended to defend the choice in an attempt to preserve self-justification.
Social factor	Ego-involving environment	The coach often developed an ego-involving environment by setting an ego-oriented goal. Such a way of thinking led the team member to focus on beating opponents. The environment was ego-involving, and the team members became ego-oriented as well. The interpersonal relationships within the team were poor.
	Growing team excitement for the upcoming match.	The team was very excited just before the match. Although the team was generally proactive and energetic, there were moments when their energy dipped. However, they showed great enthusiasm for their retirement match. Levels of nervousness in team tended to rise during the competition season.
	Responsible role in the team	The athlete was a newly appointed team leader and has high self-esteem. The athlete held a leadership position on the team and felt deeply responsible for its management. Being in a leadership position, the athlete was afraid of making mistakes and felt a strong sense of responsibility when they occurred. Despite the athlete's efforts as captain to prioritize teammates and foster a positive team environment, the atmosphere remained negative.
	Absence of coach or insufficient guidance	There were no coaches or staff to guide the athletes. The coach did not instruct either skill or tactics. Only emotional instructions were provided. The coach raised concerns about injuries, but failed to provide concrete injury prevention strategies or adequate sport-specific technical guidance. No professional guidance was provided by the coach. Instructors/trainers had no knowledge of reinjury prevention.
Situational factor (at the moment of injury)	Challenging unfamiliar situations	Athletes were playing an unfamiliar position or tactics at the time of their injury. Athlete was assigned a new position in a match. Athlete felt difficulty controlling her body in a new position.
Skill and body movement factor	Low skill levels	The athletes’ skill level was low due to a lack of professional coaching. The athlete was highly active, but her playing style relied more on physical attributes than on refined technical skills.
	Injuries during familiar or automated movements	Ironically, the movement at the time of injury was more powerful than usual, but this unexpected performance surpassed the athlete's control capacity and resulted in injury. The movement at the time of injury was one that the athlete routinely performed; it was neither particularly confident nor technically demanding. The body movement at injury was basic and automated

SCAT, Steps for Coding and Theorization.

**Table 3:** Number of themes included in the narrative data of each participant

Meta-factors		Physical		Educational	Psychological		Cognitive		Social				Situational	Skill and body movement		
Themes		Theme 1	Theme 2	Theme 3	Theme 4	Theme 5	Theme 6	Theme 7	Theme 8	Theme 9	Theme 10	Theme 11	Theme 12	Theme 13	Theme 14	Total
Participants	#1	✔	✔	✔	✔	✔		✔	✔	✔		✔		✔	✔	11
	#2	✔	✔	✔			✔	✔	✔		✔				✔	8
	#3		✔	✔			✔		✔			✔		✔	✔	7
	#4	✔	✔	✔			✔	✔			✔	✔		✔	✔	9
	#5	✔	✔	✔	✔				✔			✔			✔	7
	#6	✔		✔	✔	✔	✔	✔	✔	✔	✔		✔	✔	✔	12
	#7			✔	✔		✔			✔	✔	✔	✔	✔	✔	9
	#8	✔		✔		✔						✔		✔	✔	6
	#9			✔		✔	✔	✔				✔		✔	✔	7
	#10		✔	✔	✔	✔	✔			✔		✔		✔	✔	9
	Total	6	6	10	5	5	7	5	5	4	4	8	2	8	10	

### 3.1 Meta-factor 1: physical factor (perceived as physical-related)

This meta-factor refers to athletes’ perceived bodily condition and physical warning signs before injury, rather than to objective physiological measures alone. Although the themes included cognitive and behavioral elements, they were classified as physical-related because the narratives consistently centered on underlying physical states, such as fatigue, discomfort, instability, and other bodily signs of vulnerability. Thus, the categorization was based on the central source of injury risk described in the narratives rather than on the athletes’ subsequent interpretations or responses.

#### 3.1.1 Theme 1: the mismatch between perceived and actual physical condition

Seven out of ten athletes (7/10) reported feeling physically well at the time of injury. However, further probing revealed underlying issues such as fatigue, sleep deprivation, discomfort, and overall physical decline. These accounts were interpreted as instances in which athletes perceived themselves to be in good condition despite underlying signs of physical vulnerability. For example, athlete #10 stated, “*I felt my stamina had decreased after the examination, but I still believed my physical state was as usual.”*

#### 3.1.2 Theme 2: ignoring injury warning signs

Six out of ten athletes (6/10) were identified by coaches or medical staffs as having physical risk factors for injury, and the athletes themselves also reported bodily discomfort before injury onset. However, no preventive action was taken. For example, athlete #3 stated, “*I believed that I would inevitably get injured someday. During training, the trainer pointed out the risk of injury. I didn't take any specific measures.”* These narratives reflected situations in which physical vulnerability was recognized, but no action was taken to address it.

### 3.2 Meta-factor 2: educational factor

This meta-factor refers to limitations in athletes’ knowledge and understanding of injury mechanisms, risk management, and preventive behavior.

*Theme 3: Limited knowledge of injury and prevention.*

All the participating athletes (10/10) demonstrated a limited understanding of sports injuries and the approaches available for their prevention. Most acknowledged their injuries only after diagnosis or from external information sources. This theme points to a broader issue: “A lack of knowledge and self-regulation regarding injury prevention was evident among athletes, coaches, and trainers.”

### 3.3 Meta-factor 3: psychological factor

This meta-factor refers to athletes’ emotional and motivational states before injury, particularly those that may influence risk perception, stress responses, and decision-making in ways that increase vulnerability to injury.

#### 3.3.1 Theme 4: high-stress conditions

Half of the participants (5/10) reported experiencing substantial psychological stress before injury. This was particularly evident among athletes with a history of re-injury, who described anxiety, frustration, and pressure to return to sport quickly while also experiencing fear of movement. These narratives were interpreted as reflecting an underlying psychological vulnerability that was not fully recognized by the athletes themselves. One illustrative description was: “During the return-to-sport phase, the athlete felt nervous and fearful of fully engaging in competitive activities.”

#### 3.3.2 Theme 5: elevated motivation levels

Five out of ten athletes (5/10) reported experiencing elevated motivation before and during the competition. Although high motivation is generally regarded as adaptive, the experts discussed whether, in the present context, it could also function as a factor related to injury occurrence. This theme was retained because several narratives showed a conceptually coherent pattern linking heightened motivation with narrowed attention, reduced situational awareness, and risky play selection.

A representative example was athlete #9, who sustained an ACL injury during a contact play in a lacrosse match. At the time of injury, the athlete described being more motivated than usual, feeling in better-than-usual condition, and being strongly driven to score. This intense focus appeared to narrow attentional breadth and contributed to entry into a high-risk space during play. As the athlete stated, “*I was more fired up than usual. When the ball came to me, I was so eager and really wanted to score that my field of vision narrowed, and I ended up driving straight into a tight space.*”

Based on such narratives, elevated motivation was interpreted not as adaptive engagement per se but as a contextual state in which outcome-focused attention may override situational risk appraisal. The theme was therefore retained as a psychological factor potentially related to sports injury occurrence.

### 3.4 Meta-factor 4: cognitive factor

This meta-factor refers to athletes’ attentional focus, perceptual processing, and judgment during play at or immediately preceding injury occurrence.

#### 3.4.1 Theme 6: narrowing of attention

Six out of ten athletes reported that their field of vision had become narrower than usual at the time of injury because of intense focus during play. This attentional narrowing appeared to reduce awareness of surrounding players and situational information, thereby impairing decision-making. The theoretical interpretation of this theme was “a lack of focused attention, situational awareness, and spatial recognition.” One athlete described this process as follows: “*A strong desire to score, combined with excessive focus on personal performance, led to tunnel vision and poor decision-making during play.*”

#### 3.4.2 Theme 7: overconfidence

Five out of ten athletes (5/10) repeatedly reported overconfidence during their accounts. Athletes described experiences such as strong certainty in their predictions, confidence that their judgment at the time of injury was correct, justification of their own choices, perceived physical superiority, and heightened self-evaluation relative to others.

For example, a lacrosse goalkeeper (athlete #1) reported having strong confidence in her ability to predict shot direction and described moving preemptively based on this expectation during a shooting drill. She perceived her judgment at that moment as appropriate and stated, “*I believed my decision at the time of injury was correct.*” However, when the ball was directed toward the opposite side of her anticipated trajectory, she attempted a rapid change of direction. As a result, the athlete sustained an ACL injury. The relevance of this theme to sports injury occurrence has been addressed in the Discussion section.

### 3.5 Meta-factor 5: social factor

This meta-factor refers to interpersonal and team-contextual influences that may shape athletes’ psychological state, decision-making, and exposure to injury risk.

#### 3.5.1 Theme 8: Ego-involving environment

Five out of ten athletes (5/10) belonged to teams characterized by ego-involving climates. These environments were shaped by coaches who emphasized ego-oriented goals, such as outperforming others and winning at all costs. Theoretical insights described these social situations as: “The coach fostered an ego-involving environment, setting goals focused on competition and results. Consequently, the entire team prioritized winning.”

#### 3.5.2 Theme 9: growing team excitement for the upcoming match

Four out of ten athletes (4/10) experienced heightened excitement or nervousness before and during competitions. They reported experiencing a shared state of heightened physiological and psychological arousal in the moments leading to competition. Theoretical description includes “The team becomes energized only right before games, with nervousness intensifying during competition.”

#### 3.5.3 Theme 10: responsible role in the team

Four of the ten athletes (4/10) held key leadership roles within their teams. Across narratives, these athletes described a strong sense of responsibility, high self-evaluation, and pressure related to team management and performance. This theme was retained because these elements formed a conceptually coherent pattern suggesting that leadership-related responsibility may indirectly increase psychological and behavioral demands in contexts relevant to injury occurrence. For example, athlete #7 stated, “*I was too impatient because of my teammate. That teammate asked me to change to a position I had never played before, and I accepted it. I’m usually not impatient at all, but I was impatient at that time. When I got injured, both my mental state and the team situation were bad.*”

#### 3.5.4 Theme 11: absence of coach or insufficient guidance

Eight out of ten athletes reported a lack of proper coaching support. Neither the coach nor the available coach was present, and neither provided minimal technical instructions. The teams were student-led and operated without the support of professional coaches. Theoretical descriptions include: “There were no coaches or staff members.” “The coach was the type who refrained from providing guidance.”

### 3.6 Meta-factor 6: situational factor (at the moment of injury)

This meta-factor refers to the immediate contextual and task-related circumstances surrounding the injury event, particularly situations that impose unfamiliar or challenging demands on the athlete at the moment of injury.

#### 3.6.1 Theme 12: challenging unfamiliar situations

Two out of ten athletes sustained injuries while performing in challenging, unfamiliar situations, roles, or tactical setups. Theoretical explanations include “The position and tactic were unfamiliar” and “I had difficulty controlling my body in the new position.”

### 3.7 Meta-factor 7: skill and body movement factor

This meta-factor refers to athletes’ skill-related limitations and movement characteristics during play, including insufficient technical proficiency and injuries occurring during familiar or automated movements.

#### 3.7.1 Theme 13: Low skill levels

Eight out of ten athletes demonstrated relatively low skill levels and limited tactical variability. Theoretical interpretations include: “Players relied heavily on basic physical conditioning and high activity levels” and “Inadequate skills were apparent, possibly due to a lack of specialized coaching and overreliance on instinctive play.”

#### 3.7.2 Theme 14: injuries during familiar or automated movements (diverse perceptions of body movement quality at injury)

Regarding the body movements associated with play in which sports injuries occurred, three out of ten participants reported that the injury occurred during a movement they were particularly skilled at and routinely performed. The remaining seven participants stated that the injury occurred during a routine movement that was neither proficient nor deficient. Notably, none of the participants reported that the injury occurred during a movement they considered poor. Theoretical insights reflect that “injuries occurred during routine automatic movements.”

## 4 Discussion

This study explored the multiple factors associated with acute sports injuries through semi-structured interviews. Using the SCAT, 14 themes were identified and grouped into seven meta-factors. This study obtained insights into the detailed circumstances surrounding the occurrence of acute sports injuries that could not be ascertained from the questionnaire survey in our previous study (15). Based on the seven meta-factors, it was suggested that the sports injuries were attributed to misperceptions of physical condition, insufficient knowledge of safe play, psychological tension, suboptimal cognitive behavior, eccentric social environments, demanding play situations, and improper movement or low skill levels. Each meta-factor is discussed in detail in the following sections.

### 4.1 Physical factor (perceived physical-related)

The meta-factor of “Physical (perceived physical-related)” included two themes of “The mismatch between perceived and actual condition” and “Ignoring injury warning signs.” The theme of “The mismatch between perceived and actual condition” was consistently reflected in the athletes’ narratives. While many athletes reported being in good physical condition, further exploration revealed underlying issues, such as fatigue and localized musculoskeletal discomfort, particularly in areas like the knees. This discrepancy suggests that athletes may not fully recognize or accurately report their physical limitations, which could have implications for injury prevention and performance monitoring (22).

The tendency to ignore injury warning signs emerged as a recurrent theme among the participants. Despite being aware of these signs and receiving cautionary feedback from others, the athletes frequently failed to respond appropriately. Two underlying conditions were identified, which may help explain this behavioral pattern. First, psychological mechanisms may be at play in situations where athletes consciously avoid acknowledging potential risks. Particularly, fear of negative consequences, such as losing the opportunity to compete or being excluded from the starting lineup, may deter them from admitting to injury symptoms (23). Second, some athletes may recognize the risk but refrain from acting simply because they do not know how to respond. In such cases, inaction is likely related to a lack of knowledge or understanding.

From a physical perspective, participants described experiencing fatigue, possible overwork, lack of sleep, and decline in physical fitness, all of which collectively indicate compromised physical conditioning. This impaired conditioning itself increases the risk of sports injury. Furthermore, the failure to recognize such deterioration and to perform at one's usual level may further heighten injury risk. For these reasons, we categorized this theme as a physical factor, as the core mechanism contributing to injury risk was grounded in the athletes’ actual physical condition. Given that it is not possible to definitively determine whether the primary source of risk lies in cognitive misinterpretation or in the athletes’ physical state, we classified this theme as a Physical factor based on the consistent indications of physical deterioration evident in the athletes’ narratives.

### 4.2 Educational factor

Furthermore, the meta-factor of “Educational factor” included the theme of “Limited knowledge of injury and prevention,” which reflects a fundamental lack of understanding among athletes regarding how to engage in sports safely. This deficit appears to impede their ability to recognize injury warning signs and take appropriate preventive action. Many participants involved in sports characterized by a high injury risk did not consistently adopt preventive behaviors. This suggests that their limited knowledge may have contributed directly to the occurrence of injuries. Although knowledge alone cannot prevent all injuries, it plays a critical role in equipping athletes with the awareness required to reduce risks (24). Previous research involving collegiate athletes in the United States revealed considerable individual variation in knowledge and perceptions of injuries, such as ACL tears (24), emphasizing the importance of targeted education. Furthermore, even when athletes are aware of injury risks, a lack of knowledge regarding how to respond may have led to inaction. Previous research has shown that, while 84% of soccer players surveyed acknowledged the importance of injury prevention, they lacked adequate knowledge of effective preventive strategies (25). This finding is consistent with previous observations that athletes sometimes recognize warning signs but fail to act accordingly, likely due to uncertainty rather than deliberate inaction. Educational interventions aimed at improving injury-related knowledge may result in short-term gains; however, without reinforcement, these effects often diminish within 2 months (26). Therefore, continuous and structured educational efforts are necessary to promote lasting understanding.

### 4.3 Psychological factor

The meta-factor of “Psychological” encompassed the themes of “High stress” and “Elevated motivation levels” which captured key psychological characteristics of the participants.

The relationship between psychological stress and the occurrence of sports injuries has been well documented in previous research (7, 13). Although the specific sources of stress varied among individuals, a common theme was the experience of heightened stress and perceived time pressure, particularly during the return-to-play phase following rehabilitation. These findings, align with those of previous studies, suggest that negative life events, elevated psychological stress, and increased mental strain are associated with a heightened risk of sports injuries (27).

In this study, elevated motivation was interpreted not as adaptive engagement, but as a contextual state in which outcome-focused attention overrides situational risk assessment.

Theoretical insights suggest that heightened motivation may inadvertently increase injury risk through overexertion: Athletes expressed enthusiasm for improvement and strong aspirations for success. This example illustrates a process in which excessively high motivation led to attentional narrowing, reduced situational awareness, and ultimately to risky positional choices during play.

The rationale for selecting this theme was based on the identification of conceptual coherence among several related elements, including a strong desire to improve performance within a limited remaining time before retirement, strong motivation for personal growth, expectations of one's own performance, heightened mental readiness, and a sense of urgency stemming from impending team selection. In relation to sports injury occurrence, a highly motivated state was considered to have the potential to interfere with calm and deliberate decision-making within specific competitive contexts.

Beyond psychological stress, elevated motivation emerged as another salient psychological factor influencing injury risk. Interestingly, some participants demonstrated high levels of motivation even when their technical skills or physical awareness were insufficient. This imbalance between strong motivational drive and limited physical or technical readiness may lead athletes to train or compete beyond their actual capacities, thereby increasing vulnerability to injury. Previous studies on recreational runners have also reported that athletes with higher levels of intrinsic motivation tend to experience more injuries, although whether these injuries were acute sports injuries or overuse-related conditions was not explicitly stated (28). Although these findings were derived from recreational runners rather than competitive athletes, similar mechanisms—such as increased training load, heightened commitment to performance goals, and reduced attention to recovery—may operate across different sports and competitive contexts.

Excessive motivation may also elevate cognitive load and stress responses, particularly when athletes impose demands on themselves that exceed their current conditioning, technical proficiency, or injury prevention knowledge. Under such conditions, attentional resources may become strained, increasing the likelihood of risky decision-making, inefficient movement strategies, or overtraining behaviors that elevate injury risk.

In our interviews, participants frequently exhibited elevated motivation levels; however, their limited physical conditioning, insufficient knowledge of injury prevention, and gaps in technical proficiency appeared to create a mismatch between self-imposed performance demands and actual capabilities. This imbalance likely functions as a contextual risk factor that increases susceptibility to sports injury, particularly in competitive environments characterized by pressure to improve performance or return quickly to play.

### 4.4 Cognitive factor

The cognitive meta-factor encompassed the themes of “Narrowing attention” at the time of injury and “Overconfidence.” Six out of ten athletes reported that during the play in which the injury occurred, their attention had become so narrowly focused on their inability to remain aware of their surroundings. One athlete recalled, “*Upon reviewing the video footage afterward, I realized that the space I ran into was not as wide as I had perceived at the time. This spatial misperception leads to a collision with another player, resulting in injury.*” These observations suggest that attentional narrowing may impair spatial perception, leading to poor decision-making, such as entering inadequately sized spaces, and ultimately, sports-related injuries. Cases in which spatial misjudgment contributes to ACL injuries have been reported, particularly in team sports, such as soccer (29) and handball (30).

Narratives from athletes suggest that excessive confidence in performance, physical abilities, and decision-making during play may underlie the occurrence of sports-related injuries. One athlete recalled that even when making a decision that eventually led to injury, they felt absolute confidence in their choice. Confident play is essential for high-level athletic performance (31). Interestingly, one athlete, while acknowledging that the decision leading to the injury was inappropriate, repeatedly attempted to justify his actions as driven by the overconfidence he held at that time. In such a mental state, it becomes difficult to reflect objectively on the experience of injury, thus increasing the likelihood of repeating the same poor decisions when faced with similar situations in the future.

Moreover, previous studies have indicated that cognitive distortions are associated with the occurrence of sports injuries (32). In line with this finding, the athletes’ narratives suggest that excessive confidence may contribute to such cognitive distortions, thereby increasing the likelihood of sports-related injuries. Additionally, it is conceivable that overconfidence may lead athletes to choose risky actions or plays that carry a higher probability of injury. Overconfidence appeared to reduce athletes’ sensitivity to potential risks and to promote the selection of actions or plays with a higher probability of injury.

From a theoretical perspective, within the Risk Structure Model of sports injury occurrence proposed by Ogasawara et al., sports injuries are understood to arise when an athlete's psychological state (such as impatience or tension), as well as their cognition and perception, lead to judgment errors (18, 19). Within this conceptual framework, overconfidence can be interpreted as a psychological factor that distorts risk perception and decision-making, thereby increasing the probability of selecting dangerous or suboptimal actions. Taken together, the present findings extend existing research by illustrating, through athletes’ subjective narratives, how overconfidence functions not merely as a general psychological trait but as a contextual risk factor operating within specific competitive situations. Consequently, overconfidence could serve as a contributing factor to the occurrence of sports injuries.

### 4.5 Social factor

From the participants’ narratives, four major themes emerged as social factors contributing to their injuries: “Ego-involving environment,” “Growing team excitement for the upcoming match,” “Responsible role in the team,” and “Absence of coach or insufficient guidance.” These factors are not mutually exclusive but appear to be deeply interrelated and mutually reinforcing. Most participants reported that their coaches or teams fostered an ego-involving environment by emphasizing outcome-based goals such as defeating opponents, mastery, or skill development. This environment encourages intra-team competition and nurtures ego-oriented thinking among athletes. Such a climate, characterized by low-quality interpersonal relationships, aligns with the concept of an ego-involving motivational climate described in the Achievement Goal Theory, which has been reported to be associated with negative effects, ill-being, and extrinsic forms of motivation (33). This ego-oriented atmosphere intensified in the period leading up to high-stakes matches, such as final or retirement games. During this period, teamwide excitement and nervousness increased significantly, further heightening the emphasis on winning. As emotional arousal rises collectively across teams, individual players may become more willing to take physical risks in pursuit of team success. Compounding this, many participants reported an absence of professional coaching or a lack of effective guidance. These findings suggest that a lack of appropriate coaching may have amplified the ego-involving climate by failing to offer counterbalancing mastery-oriented frameworks. Moreover, as discussed in the “Educational factor” section, the lack of guidance meant that athletes had limited access to information about injury prevention strategies or safe training practices. In such a high-pressure and poorly guided environment, athletes in responsible team roles, such as captains or newly appointed leaders, reported experiencing a heightened psychological burden. These individuals often prioritized team harmony and success over personal well-being and expressed a strong fear of making mistakes. Consequently, they may have engaged in behaviors that compromised their safety, such as playing through pain or overtraining, due to the perceived expectations of leadership. This mechanism is consistent with the stress–injury model, which posits that athletes occupying responsibility-laden roles experience elevated stress levels that, through heightened psychophysiological stress responses, increase injury risk (7). The present findings extend this model by demonstrating how social roles within teams interact with motivational climates to amplify stress-related injury risk.

Within the Japanese cultural context, where interdependent behavioral norms are often emphasized, this heightened sense of responsibility may further intensify athletes’ need for approval. Such approval-seeking tendencies may increase the likelihood that athletes in leadership roles push themselves beyond their physical limits. These findings are consistent with previous studies on Japanese athletes, which have shown that a strong desire for approval from coaches is associated with greater severity of sports injuries (15). Such approval-seeking tendencies may be reinforced by culturally embedded interdependent behavioral norms, which are guided not by personal preferences but by expectations regarding others’ behavior (15, 34).

### 4.6 Situational factor

The meta-factor of “Situational factor” holds the theme of “Challenging unfamiliar situations.” Although the number was small, two participants (athlete #6 and #7) sustained injuries while playing in positions that differed from their usual positions. Notably, one of them requested a position change because of fatigue and difficulty continuing to play. It is reasonable to assume that playing with unfamiliar positional demands may compromise motor control. These circumstances may significantly increase injury risk (29).

### 4.7 Skill and body movement factor

The meta-factor of “Skill and body movement factor” included the themes of “Low skill levels” and “Injuries during familiar or automated movements.” The athletes who participated in this study perceived their limited skill levels as a contributing factor to their injuries. They attributed this lack of proficiency to the absence of consistent access to professional instruction, which they believed hindered the development of sports-specific skills. Although it is impossible to objectively evaluate athletes’ actual skill levels based solely on their narrative accounts, their reflections suggest a perceived link between skill limitation and the occurrence of injury. One athlete stated that her injury was linked to a playing style that relied excessively on physical strength rather than technical skills. She further recalled that the body movement at the time of the injury was unusually forceful and more powerful than her typical performance, and beyond what she had anticipated. This implies that compensating for technical deficiencies with heightened physical exertion may result in a performance that surpasses the athlete's physical capacity, subsequently elevating injury risk. Previous studies reported that underdeveloped motor and tactical skills may contribute to injury risk (35), which aligns with the athletes’ perspectives in this study. Intriguingly, many athletes have reported that their injuries occur during familiar and automated movements. To our knowledge, the fact that routine, low-complexity actions, typically not demanding in terms of motor control, are associated with injury, has not been previously documented. Although injuries under high physical and cognitive demands are well established and expected, the occurrence of injuries during low-stress (29, 36) habitual movements raises additional concerns regarding injury prevention. Therefore, even during routine movements, it is essential to maintain awareness of neuromuscular control, highlighting the need to consciously regulate movement in daily contexts as part of injury prevention strategies.

### 4.8 Limitations

Several limitations should be acknowledged. First, although the present study reached semantic saturation within our study sample, the number of participants was small and several cases involved similar injury types, particularly knee injuries. Therefore, the findings should be interpreted as context-specific and not as representing the full range of sports injury experiences. Future research should include a larger and more diverse sample to examine the transferability of these findings across injury types, sports, and competitive contexts.

Second, this study focused exclusively on acute sports injuries. Accordingly, the findings help clarify the context of acute injury occurrence, but they cannot be directly applied to chronic injuries, which may involve different mechanisms.

Third, as most participants were involved in team sports, careful consideration is required when applying these findings to individual sports or aesthetic disciplines, where situational and social contexts may differ substantially.

Finally, member checking was not conducted because a substantial period had elapsed between the interviews and completion of the analysis, making participant follow-up difficult owing to changes in affiliation and concerns about participant burden. This should be recognized as a methodological limitation of the present study.

## 5 Conclusion

By conducting interviews with athletes, this study helped clarify the context in which sports injuries occur, and the physical, psychological, situational, and social factors that may contribute to sports injuries were investigated. These findings suggest that a lack of knowledge and awareness, maladaptive cognitive functions, elevated motivation levels under stressed conditions, and fragile team conditions underlie sports injuries. Addressing these multifactorial issues may contribute to injury prevention.

## Data Availability

The original contributions presented in the study are included in the article/Supplementary Material, further inquiries can be directed to the corresponding authors.
