# Supplementary material for: Multifactorial background of acute sports injuries in young Japanese athletes—a semi-structured interview study
Source: Front Sports Act Living. 2026 May 26;8:1813583. doi: 10.3389/fspor.2026.1813583 (PMC13245997; doi:10.3389/fspor.2026.1813583)
Supplement: Supplementary file 1 [file Table1.docx]

**Supplement Table S1. Representative Example of SCAT Coding from an Athlete’s Narrative**

| **Question** | **Raw narrative** | **Step 1** | **Step 2** | **Step 3** | **Step 4** |
| --- | --- | --- | --- | --- | --- |
| Please tell us your age, sex, sport, and competitive level. | My name is XX. I’m 22 years old, and I’m female. I used to belong to the lacrosse team. My position was goalkeeper. I played lacrosse for four years, from my first year to my fourth year at university. Before that, I had played softball since junior high school, and I also played handball in high school. As for my competitive level, I have experience competing in regional tournaments. | 22 years old, female, lacrosse, goalkeeper, 4 years, softball, handball, regional tournaments | age 22, woman, field sport, goalie, four-year experience, prior ball-sport background, regional-level competition | A position unique within the team; directly involved in goals conceded. | Goalkeeper position |
| Please tell us the injured body part and your injury history. | This time, the injury was a right ACL tear. I got injured in July 2021. I haven’t had surgery yet. As for my injury history, I tore my left ACL in high school. I had reconstruction surgery for that one. That happened in the spring of my second year of high school, so about four years had passed between that injury and this one. Back in high school, I tore it while playing handball during practice. Both injuries were non-contact injuries. In both cases, I guess I was putting weight more on the front of my foot, around the ball of the big toe, and then tore it when I changed direction. | right ACL tear, July 2021, no surgery yet, left ACL tear, reconstruction surgery, 4 years earlier, handball practice, non-contact, forefoot loading, ball of the big toe, change of direction | right anterior cruciate ligament rupture, injury onset in 2021, untreated surgically, prior contralateral ACL rupture, reconstructive procedure, four-year interval, practice setting, noncontact mechanism, front-foot weight bearing, metatarsal-head loading, cutting maneuver | Four years earlier, the athlete had sustained a left ACL tear and underwent surgery. This time, she injured her right ACL during a cutting movement (non-contact). | Repeated severe knee injuries |
| At that time, were there any positive or negative events on your mind? | As for negative things, when I was in my third year of university, I was really worried about whether I should start job hunting or go on to graduate school. I just couldn’t make up my mind about my future. | negative event, worried about future, job hunting, graduate school, couldn’t decide, career path | adverse life event, uncertainty, employment decision, postgraduate study, indecision, vocational direction | Anxiety about the future; self-denial. | Anxiety related to life decisions; anxiety about career path; feelings of self-denial; anxiety. |
| Please tell us about the injury: when it happened, whether it occurred during a game or practice, and if it was during a game, how important the game was. | The injury I had in university happened on July 3, 2021, and the one in high school happened in the spring. Both of them happened during practice. The university injury happened during a practice session that usually lasts about three hours, and I think it was around halfway through, so probably a little over an hour in. I think the high school injury also happened in the middle of practice. | July 3, 2021, spring, both during practice, 3-hour practice, halfway through, a little over an hour in, middle of practice | injury date, spring season, training context, mid-session, approximately one hour into practice, practice midpoint | The injury occurred in the middle of practice. |  |
| Please describe the situation when the injury occurred (looking back and recreating it as if you were playing). | I got injured during lacrosse practice. I was practicing as a goalkeeper. The drill was specifically for goalkeepers — shooters kept taking shots one after another, and the goalkeeper had to keep responding to them. When one particular shooter took a shot, I predicted from her shooting form that the ball was going to come to my right side. But in reality, the trajectory looked like it was going to the left, so when I tried to plant and change direction quickly with my foot toward the opposite side from what I had expected, I heard a popping sound in my knee. | goalkeeper drill, repeated shots, shooter’s form, predicted right side, ball went left, opposite direction, quick plant, sudden cutback, popping sound in knee | goalkeeper-specific drill, consecutive shooting, shot cue, anticipated rightward shot, leftward trajectory, reverse response, abrupt redirection, forced deceleration, audible knee pop | My prediction was wrong. I tried to do something about it. I forced myself to move. | Prediction error; forced response; did not give up; error in decision criterion. |
| Was there anything unusual about the situation when the injury occurred? | Usually, we practiced mainly on the dirt field out of the two fields we had, but sometimes we practiced on the artificial turf. The day I got injured just happened to be one of the turf practice days. Also, I had just bought a new pair of cleats. Those new cleats had really strong grip, and I think the fact that they were catching on the artificial turf was one physical aspect that was different from usual. My memory is a little vague, but I think it was actually the first day I had used those new cleats on the artificial turf. I feel like it was probably a situation that put more load on my body than usual. | usually dirt field, artificial turf that day, new cleats, strong grip, cleats catching, first time using them on turf, more load than usual | usual dirt surface, turf session, new spikes, high traction, shoe-surface interaction, first turf use, unfamiliar setup, increased mechanical load | An environment different from usual (ground condition, shoes). Felt that it increased the load. | New environment; unfamiliar equipment; maladaptation to a novel extrinsic factor; novel extrinsic factor. |
| What were you feeling when the injury occurred? | In lacrosse, there’s basically only one big tournament across the whole year — what we call the league competition. That starts around mid-August or September, so practice in early July was really about improving myself for that tournament. At the time, I was in the top year, and I guess — I wouldn’t call it panic exactly — but looking back now, I think I was more worked up than usual because I really wanted to improve. | only one big tournament, league competition, early July practice, wanted to improve, top year, more worked up than usual | single major event, league season, pre-competition practice, strong drive for improvement, final-year status, heightened tension | There are only limited opportunities. As a final-year student, I wanted to produce results. | Impatience about limited opportunities; wanting to improve performance within limited time; high-stress condition; disharmony; telling oneself “I’m not impatient”; a situation that inevitably creates impatience; a situation likely to induce impatience. |
| How was your physical condition when the injury occurred? | I felt like I was in pretty good shape. Since we were on the artificial turf, compared with when we practiced on the usual dirt field, I felt like I could move more sharply, like snap-snap. The cleats had just the right amount of grip, so even during warm-up I had the feeling like, “Today’s one of those days when I can really move well.” I think I was in pretty good condition. | pretty good shape, artificial turf, sharper movement, good grip, during warm-up, “today I can really move well,” good condition | good physical state, turf surface, quicker movement, optimal traction, warm-up phase, moving well today, favorable condition | A feeling of being in good condition. Felt that the grip allowed sharp movement. | Awareness of being in good condition; awareness of good condition under a novel extrinsic factor. |
| What was your mental state like when the injury occurred? | Mentally, I’d say I felt good. I was really fired up about the competition. I had this strong feeling like, “I’m going to control the game myself.” | felt good mentally, fired up, competition, strong feeling, “I’m going to control the game myself” | positive mental state, highly aroused, match-focused, strong determination, “I’ll take over the game” | Highly aroused state; self-responsibility. | Highly aroused state; self-perceived good mental condition; excessive sense of responsibility; overconfidence. |
| What was the team situation like, and what was your role within the team? | It was about one month before what would be our first league competition as the senior class, so I think there was definitely this team atmosphere of “Let’s really push from here.” I was in the top year, too. In lacrosse, goalkeepers usually don’t get substituted much during games. Field players run around for the whole 60 minutes, so substitutions happen a lot there, but the goalkeeper often handles the whole game alone. The younger goalkeeper hadn’t really been developed enough yet, so I think I was in the position where I was expected to play in the league, where I had to be the one out there. | one month before league, team atmosphere, “let’s really push from here,” top year, goalkeeper rarely substituted, younger goalkeeper not ready, expected to play, had to be the one | one month before competition, motivated team climate, push toward the season, senior status, fixed goalkeeper role, junior underdevelopment, expected starter, sole responsibility | The team was in a highly excited state as competition approached. Anxiety about the junior player’s development. Fulfilling one’s responsibility. Strong determination. | Team excitement; anxiety about the junior player’s development; overconfidence; lack of trust in the junior player; monopolization of responsibility; egoistic. |
| What did your supervisor or coach say to you? | Nothing in particular. There wasn’t a goalkeeper-specific coach, so we just had the team’s head coach. It’s not like the coach was telling me on a daily basis, “Do this” or “Do that.” | nothing in particular, no goalkeeper-specific coach, only head coach, no daily instructions | no specific advice, no specialist coach, only general coach, no routine direction | The coach did not actively instruct; likely no technical coaching. | Lack of specialized technical instruction; extremely low level of interaction with the coach; no pressure from the coach; no stress. |
| When the injury occurred, how wide was your visual field and attentional focus? | As a goalkeeper, you move while watching the shooter’s form, and the goalkeeper drill where I got injured was also a drill where I was taking a lot of shots. As for how wide my visual field was, I’d say it wasn’t really different from usual, but I think I was very focused. I don’t think my vision had narrowed too much. | watching shooter’s form, goalkeeper drill, lots of shots, visual field not different from usual, very focused, vision not narrowed too much | reading shooting form, goalkeeper practice, repeated shots, usual field of view, high concentration, no marked tunnel vision | Watching the shooter’s form and moving accordingly. High concentration. Acknowledged that the visual field had not narrowed too much. | Error in information processing involving prediction. Self-perception that the visual field was not overly narrowed. |
| Compared with usual, was your conditioning better or worse before the injury? | I had this feeling like, “Yeah, today’s a good day.” The way practice usually went was that we all warmed up together, then the goalkeepers split off, called over one shooter, did a goalkeeper-only warm-up, and then joined the full team drill. On days when I feel like, “Yeah, I’ve got it today,” it’s things like feeling lighter in each movement during warm-up. I guess breakfast that morning can matter too, but I felt less out of breath. Then once the warm-up was over and we started the goalkeeper-specific drill, there were things like how well I could read the shooter’s trajectory, whether I could catch and stop the shot cleanly, or whether the ball just happened to hit my body and get stopped. Around that time, during that period, I was definitely having more and more saves where I was really reading the ball trajectory and stopping the shot, so it was also a time when I felt like I was improving. I felt like my body use and my ability as a goalkeeper were really starting to come together. | “today’s a good day,” warm-up, lighter movement, less out of breath, read shooter’s trajectory, more saves, improving, body use, goalkeeper ability | good-day feeling, warm-up phase, light body feel, reduced breathlessness, reading shot trajectory, increased successful saves, improvement phase, body control, goalkeeper skill | Awareness, on the day of injury, that both condition and ability had improved. | Subjective awareness of being in good condition (both that day and during that period); awareness of improvement based on results (number of successful saves). |
| Why did you choose the play that led to the injury? | The main reason was that the ball came in the opposite direction from what I had expected. That kind of thing happens all the time, and the movement that caused the injury — cutting back off the ball of my foot — was also something I probably did dozens of times every day. | ball came opposite to expectation, happened all the time, cut back off the ball of the foot, movement done dozens of times every day | unexpected ball direction, routine occurrence, forefoot cutback, habitual movement, repeated many times daily | My prediction was wrong, and I tried to catch up right away. I didn’t give up. | Response to an unexpected event. |
| Was there anything you were feeling or concerned about regarding the injury? | Not really. I didn’t think I was going to get injured. | not really, didn’t think I was going to get injured, no concern, no expectation of injury | none in particular, no anticipation of injury, no worry, no expectation of being hurt | No recognition of warning signs of injury. | No recognition of warning signs of injury. |
| During that play, what was the positional relationship between you and the opposing player, or with your teammates, the ball, or equipment? | At that time, it was a situation where the shooter was running in about two steps from the 11-meter line to take a long shot, so the shot was taken from about 8 or 9 meters away. Once I realized my knee had gone, I lost my balance. But it wasn’t like a game situation where I had no idea when the shot was coming. I knew the shot was coming, so I think I was properly set and in a balanced stance. | 11-meter line, two steps in, long shot, 8 or 9 meters away, knew the shot was coming, properly set, balanced stance, lost balance after injury | shot from 8–9 meters, predictable shot timing, prepared stance, stable setup, post-injury loss of balance | There was a fixed assumption in the prediction. | Excessive confidence in the prediction. |
| What movement did you actually perform? Please recreate it. | It was really a goalkeeper movement. Let me think. I had both feet about shoulder-width apart, I was holding my lacrosse stick, and I was set in the stance that felt easiest for me to move from. I was set expecting the shot to come to my right, but when I saw the ball trajectory and realized it was going left, I made a pretty forced foot movement to bring my body over to the left. I got injured when I pushed off with my right foot — like kicking to the right in order to move my weight to the left. (When I filled out the questionnaire, there was a question asking if I could provide a video — I do have one.) | shoulder-width stance, lacrosse stick, ready position, expected right, saw left trajectory, forced foot movement, moved body left, pushed off with right foot, injured | set stance, stick in hand, prepared posture, anticipated rightward shot, recognized leftward flight, forced lower-limb response, shifted body left, right-foot push-off, injury event | How the body was moved after the prediction turned out to be wrong. | Excessive confidence in the prediction; prediction based on a fixed assumption. |
| Was the movement during which you got injured one you were good at, or one you were not good at? | Yeah, I think it was a movement I was good at. There are different types of goalkeepers, but I had experience in ball sports before, and I was relatively good at making saves. Reading the shooter’s form and then having the ball come the other way — that kind of thing was really routine for me. And I had trained that reaction ability for three years. | movement I was good at, ball-sport experience, good at saves, reading shooter’s form, opposite-direction ball, routine, trained reaction ability for three years | skilled movement, prior ball-game background, strong saving ability, reading shot cues, reverse-direction response, habitual situation, three years of reaction training | Pride in being good at the movement. | Pride in being good at the movement. |
| What do you think caused the injury? | I think the biggest thing is that I’d already had the same injury in high school, and what the rehab people told me was that I was moving in ways that put a lot of load on my knee ligament without really being aware of it. I think the biggest cause is probably the way I use my body — my legs, my knees. For example, when I squat, my knees tend to cave inward, going inside the line from the hip down through the leg, and that happens pretty often when I’m not paying attention. In high school, when I cut back to the right, I think I planted and changed direction off my left foot, and my knee caved inward too much and then it popped. I think the same thing happened in university too — my knee probably went inward. | same injury in high school, rehab people said movement put load on knee ligament, body use, leg use, knee use, knees cave inward, not paying attention, cut back, planted, knee went inward | prior identical injury, rehabilitation feedback, ligament-loading movement, movement pattern, lower-limb mechanics, knee control, dynamic valgus, lack of conscious control, cutting maneuver, planting action, inward knee collapse | Knee angle when not consciously controlled. | Postdiction; concern about a subjectively recognized intrinsic factor. |
| Do you think you were unlucky? | Honestly, I do think luck was part of it. Subjectively, I sometimes think that if it hadn’t been for that one shot, I might have finished the whole day’s practice without getting injured. So in the end, I do think I was unlucky too. | luck was part of it, that one shot, might have finished practice without injury, unlucky | chance played a role, single shot event, could have finished practice uninjured, bad luck | Dependent on luck. | Avoidance of responsibility. |
| Do you think there was any background reason behind your injury? | I think, like I said before, it was partly the poor way I use my knees. Also, because I had a good feel for ball sports, I preferred ball-based practice over training to improve my physical abilities. I liked drills with the ball, but I didn’t really like training that was just for building the body, like weight training or running. I did do the things that were assigned in practice and the things that were set up for individual training, and we also had student trainers who told us every time what we should be focusing on during strength training. For example, if the proper way to do a squat was to engage the glutes, I might end up putting the effort into my thighs instead. I have a sense that I was just getting through the training in the way that felt easiest for me, without really locking in on what I should have been focusing on. So I think it was both the bad way I use my knees and the fact that I still didn’t have enough muscle, and that my ligament just couldn’t tolerate it. | poor knee use, good feel for ball sports, preferred ball-based practice, didn’t like weight training or running, student trainers gave instructions, squat focus wrong, did training in the easiest way, not enough muscle, ligament couldn’t tolerate it | poor knee mechanics, confidence in ball-sport sense, preference for skill-based practice, dislike of physical training, trainer guidance, incorrect squat focus, defaulted to the easiest movement pattern, insufficient strength, tissue load intolerance | Fixation on one’s own way of doing things. | Concern about intrinsic factors and contradiction; excessive confidence. |
| Before the injury, did you think you were going to get injured? | No, I didn’t. | no, didn’t think so, no expectation of injury | no anticipation, no assumption of injury, no expectation of being hurt | No recognition of warning signs of injury. | Lack of risk awareness; insensitivity to risk. |
| Do you think the injury could have been prevented, or can be prevented? Why? | I think it could have been prevented. Earlier I said luck was part of it, but when I was in high school, I was told that the way I use my knees was bad, and I kind of just thought, “Oh, okay,” and didn’t take it that seriously. I also wasn’t consciously paying attention to how I use my knees in daily life, and I hadn’t really done strength training specifically to prevent injury. So I think it could have been prevented if I had paid more attention on a regular basis, done more conscious strength training to avoid injury, and been more aware that the way I use my body is poor — especially by paying attention to how I use my legs. | could have been prevented, told in high school knee use was bad, didn’t take it seriously, didn’t pay attention in daily life, no injury-prevention strength training, more regular attention, conscious strength training, body use, leg use | preventable, earlier warning about poor knee mechanics, dismissed advice, little daily awareness, no preventive strengthening, regular monitoring, intentional strength work, movement pattern awareness, lower-limb use | Injury can be prevented through strength training. | Self-contradiction; lack of knowledge; lack of information. |
| What do you think you will do from now on? If you were to give advice to others, what would you say? | There was usually about one girl in each year who tore her ACL, so if I were really going to give advice, I’d say this: when the trainer tells you what to focus on during strength training, pay attention to that. Don’t just go through the motions of strength training — think about what exactly you’re training, whether you’re doing it to prevent injury or to improve performance. I’d say you need to do strength training while thinking about what it actually means. | one girl each year tore her ACL, advice, trainer tells you what to focus on, pay attention, don’t just go through the motions, think about what you’re training, prevent injury, improve performance, meaning of strength training | frequent ACL cases, recommendation, trainer’s points of emphasis, attend carefully, avoid mindless repetition, consider the training target, injury prevention, performance enhancement, purpose of strengthening | Injury can be prevented through strength training. | Lack of knowledge. |
| What do you consider important in your sport? What experiences made you feel that way? | What I valued was never compromising in my day-to-day effort. That was just my personality — if I didn’t do that, I couldn’t feel satisfied. For example, before practice, I would compare my own video from the previous day with video of Olympic athletes and make myself aware of the differences before starting practice. Or from the warm-up, I had this attitude of, “I’m not going to lose to anyone.” What I valued was the desire to grow more than anyone else, in the same environment and in the same amount of time. | never compromising day to day, personality, compare my video with Olympic athletes, notice differences, before practice, from warm-up on, “I’m not going to lose to anyone,” desire to grow more than anyone else, same environment, same amount of time | daily non-compromise, achievement-oriented personality, comparison with elite athletes, awareness of gaps, pre-practice review, competitive mindset from warm-up, “I won’t be beaten,” stronger growth drive than others, same conditions, same time frame | Comparison with others; ego orientation; misunderstanding of the meaning of warm-up. | Ego orientation |

Note that the original language was Japanese. Raw narrative entries are translated into natural, conversational English to preserve the athlete’s original tone and nuance.
